# Biocatalytic Approach for Direct Esterification of Ibuprofen with Sorbitol in Biphasic Media

**DOI:** 10.3390/ijms22063066

**Published:** 2021-03-17

**Authors:** Federico Zappaterra, Maria Elena Maldonado Rodriguez, Daniela Summa, Bruno Semeraro, Stefania Costa, Elena Tamburini

**Affiliations:** 1 Department of Life Sciences and Biotechnology, University of Ferrara, Via L. Borsari, 46, 44121 Ferrara, Italy; zppfrc@unife.it (F.Z.); smmdnl@unife.it (D.S.); tme@unife.it (E.T.); 2 Department of Biotechnology Engineering of the RRNN, Salesian Polytechnic University, Av. 12 de Octubre y Wilson, Quito 170109, Ecuador; mmaldonado@ups.edu.ec; 3 GATE SRL, Via L. Borsari, 46, 44121 Ferrara, Italy; bsemeraro@gategreen.it

**Keywords:** ibuprofen, sorbitol, esterification, porcine pancreas lipase, prodrug

## Abstract

Ibuprofen is a nonsteroidal anti-inflammatory drug (NSAID) introduced in the 1960s and widely used as an analgesic, anti-inflammatory, and antipyretic. In its acid form, the solubility of 21 mg/L greatly limits its bioavailability. Since the bioavailability of a drug product plays a critical role in the design of oral administration dosage, this study investigated the enzymatic esterification of ibuprofen as a strategy for hydrophilization. This work proposes an enzymatic strategy for the covalent attack of highly hydrophilic molecules using acidic functions of commercially available bioactive compounds. The poorly water-soluble drug ibuprofen was esterified in a hexane/water biphasic system by direct esterification with sorbitol using the cheap biocatalyst porcine pancreas lipase (PPL), which demonstrated itself to be a suitable enzyme for the effective production of the IBU-sorbitol ester. This work reports the optimization of the esterification reaction.

## 1. Introduction

Ibuprofen ((R,S)-2-(p-isobutylphenyl)-propionic acid) is a traditional nonsteroidal anti-inflammatory drug (NSAID) [1] that was developed in the late 1960s [2] for the treatment of symptoms caused by arthritis such as swelling, pain, and stiffness [3]. Ibuprofen, like other nonsteroidal drugs such as ketoprofen and flurbiprofen, is widely used for its analgesic, anti-inflammatory, and antipyretic properties [4]. It is used for mild-to-moderate pain such as dysmenorrhea, headaches (including migraine), dental pain, postoperative pain, and pain caused by musculoskeletal/joint disorders, including osteoarthritis, rheumatoid arthritis, and ankylosing spondylitis [5,6]. The mechanism of action of ibuprofen for different therapeutic purposes is well established. Ibuprofen is a non-selective reversible inhibitor of cyclo-oxygenase isozyme (COX)-1 and COX-2, which are responsible for the conversion of arachidonic acid into prostaglandins, including thromboxane and prostacyclin [7]. Due to its chiral center, ibuprofen has two enantiomers [8]. However, it is well documented that the therapeutic activity of ibuprofen is mainly attributable to the (S) enantiomer, which is 160 times more effective than the (R) enantiomer [9]. It has been reported that, in the human body, (R) ibuprofen can undergo “metabolic inversion” to produce (S) ibuprofen [10]. Due to its high patient compliance, cost-effectiveness, reduced sterility constraints, and flexibility, the most common route of administration for ibuprofen is the oral [11] via tablets, caplets, or capsules at 200, 400, or 800 mg strengths [12,13]. The dose is 200–400 mg (5–10 mg/kg in children) every 4–6 h for a maximum of 1.2 g per day in adults [14]. Due to its aqueous solubility of 21 mg/L [15], ibuprofen is a poorly water-soluble drug, characterized by dissolution-limited oral bioavailability [16]. The low rate of dissolution from the currently available solid dosage forms, and the consequent poor bioavailability from a high oral dose [17], can cause severe unwanted adverse effects. The chronic use by oral administration of a NSAID may cause gastric mucosal damage—more concretely, stomach ulceration, bleeding, and perforation [18].

Poorly water-soluble drugs present ongoing challenges with their translations into viable medicinal products [19]. More than 40% of new chemical entities (NCE) synthesized by combinatorial screening programs and possessing superior pharmacological activities are poorly soluble, which is a great obstacle in formulation development [20,21]. Thus, drugs with poor water solubility require novel formulation approaches to improve their rates of dissolution and oral bioavailability [22]. There are formulations with ibuprofen salt conjugates (e.g., arginate, lysinate, and sodium) in an attempt to enhance dissolution rates in the stomach [23]. Ibuprofen’s solubility is limited in the stomach because it is a carboxylic acid in an aqueous acidic media [24]. For this reason, one way to increase the water solubility of ibuprofen could be an esterification reaction between its carboxylic acid and an alcohol to obtain an enhanced water-soluble prodrug designed to effect better oral availability [25]. A prodrug is a compound that can undergo biotransformation before exhibiting its pharmacological properties [26]. As early as 1980, a toxicological and pharmacological study of several derivatives and formulations of ibuprofen showed the prodrug ibuprofen guaiacol ester as a suitable form of this drug decreasing toxicity in rats [27]. The administration of four brain targeting L-ascorbic acid prodrugs of ibuprofen exhibited increased levels of ibuprofen in brain [28]. Furthermore, the N,N-disubstituted aminoalcohol ester of ibuprofen resulted in a significant reduction of ulcerogenicity in the stomach [29].

Canonically, the esterification reaction was performed chemically by a Fischer esterification [30]. Instead of chemical modifications, biotechnological approaches can represent green ways for the synthesis of bioactive derivatives of ibuprofen [31,32]. Green processes must use eco-friendly, non-toxic, reusable catalysts to synthesize valuable chemical compounds [33]. As implied above, ibuprofen is a poorly water-soluble drug. Thus, its esterification with hydrophilic compounds can achieve molecules enhanced in water solubility and consequent bioavailability. This strategy can be used by a biocatalyzed esterification reaction. For this purpose, lipases represent the most used biological tool for enzymatic esterification. Since the nineties, lipases (triacylglycerol hydrolases, EC 3.1.1.3) were used as biocatalysts in industry due to their activity under mild reaction conditions, the lack of need for co-factors, their synthetic activity in non-aqueous solvents, and their wide range of substrate stereo-specificities [34]. Lipases are widely used as the biocatalysts for esterification or transesterification reactions, and they exist in free or immobilized form [35]. Among the most commonly used non-immobilized (free) lipases for esterification or transesterification reactions are those from the microorganisms *Rhizomucor miehei* [36], *Candida. rugosa* [37], and *Pseudomonas cepacian* [38]. Immobilized lipases such as Novozyme 435 (*C. antarctica* lipase B, CALB) [39], Lipozyme RM-IM (lipase from *R. miehei*), and Lipozyme TL-IM (lipase from *T. lanuginosus*) [40] are also commercially available. However, lipases commercially available are also derived from pigs (porcine pancreas lipase, type II; PPL) [41] or horses (horse pancreatic lipase) [42]. PPL is reported to be more advantageous than the microbial lipases in the organic environment due to its high selectivity, great tolerance to solvents, high catalytic activity, and thermal-stability at a high temperature under low water concentrations [43,44]. Porcine pancreatic lipase (PPL) has been employed as a catalyst for the esterification reaction between the hydroxyl group of lactic acid and the carboxylic group of organic acids, providing high bench-level conversational yields [45].

Several works on lipase-catalyzed profen esterification in organic media are available [46]. Ibuprofen has been enzymatically esterified with glycerol in solventless-media and the kinetic model was reported [47]. The ascorbic acid ester of the NSAID flurbiprofen has been synthesized by lipase-catalyzed transesterification and esterification [48]. Furthermore, S-naproxen esterification has been irreversibly performed on dimethyl carbonate by biocatalysis [49]. Furthermore, PPL was used in the optical resolution of (R, S)-ibuprofen in methanol through enantioselective esterification. This lipase showed a preference for catalyzing the esterification of S-(+)-ibuprofen [50].

Some lipases, such as PPL, undergo interfacial activation due to an opposite polarity between the enzyme (hydrophilic) and their substrates (lipophilic). The reaction occurs at the interface between the aqueous and the oil phases where a conformational change associated occurs and increases the enzymatic activity. Hence, interfaces are the key spots for lipase biocatalysis and an appropriate site for modulating lipolysis activity [51]. Hence, the use of an aqueous-organic solvent biphasic system can produce interfacial activation of the lipases. To enhance the surface of the interface, vigorous mixing of the two phases forms a suspension with a significantly large interfacial area. This system produces advantages, such as better solubility of substrates and product, shifting of thermodynamic equilibria (synthesis takes place instead of hydrolysis), the potential of enzymes to be used directly within a chemical process, and the possibility of solubilization of hydrophilic and lipophilic compounds [52]. Emulsions generated by the mixing of two phases have been exploited for the o/w hydrolysis of tributyrin by *Thermomyces lanuginosus* lipase (TLL) [53].

Moreover, the activity of a biocatalyst in mostly organic reaction mixtures is usually greatly affected by the level of water. To better understand the impacts of water in biocatalyzed reactions, it is important to consider the thermodynamic water activity (a_w_), in addition to water content. The water activity governs the degree of hydration of the enzyme and gives a direct indication of the mass action of water [54]. Water activity has been shown essential in lipase-catalyzed reactions in non-aqueous solutions and it can significantly impact the catalytic activity of lipases [55]. Water is known to act as a molecular lubricant for lipase in the non-aqueous media, whereas if the water layer is stripped away, the active site of the lipase might show conformational changes and thus display lower catalytic activity [56]. It is essential to add a certain amount of water to maintain the stability and flexibility of enzymes in the organic solvent. Thus enzyme-bound water is essential for catalysis and serves as a lubricant for the enzyme [57]. However, biphasic systems that contain large amounts of water allow adequate hydration of the biocatalyst. Recently, Bracco et al. have shown that a_w_ is a suitable parameter to distinguish between lipases and esterases [58]. Although some lipases (such as those derived from CALB and *R. arrhizus*) maintain good activity at low water content, other lipases require higher water activity levels to show good catalytic activity—e.g., lipase from *P. cepacian* [59]. Furthermore, it has been reported how alcohol and solvents can affect the lipase-mediated esterification for the racemic resolution of ibuprofen [60]. For this reason, our study designed a strategy for effective esterification of highly hydrophobic ibuprofen and highly hydrophilic sorbitol in a biphasic system.

Sorbitol ((2R,3R,4R,5S)-hexane-1,2,3,4,5,6-hexol), less commonly known as glucitol, is a polyalcohol with a sweet taste and is slowly metabolized by the human body. It can be obtained by reduction of glucose, which changes the converted aldehyde group to a primary alcohol group. Most sorbitol is made from potato starch, but it is also found in nature, for example, in apples, pears, peaches, and prunes [61]. Sorbitol is often used in modern cosmetics, as a humectant and thickener, and as a cryoprotectant additive in food [62]. It is one of the preferred sweetening agents, as it has low caloric content and a low glycemic index value (about one-half of that of sucrose) and it is free from any possible carcinogenic effects. Another advantage that sorbitol offers is related to its resistance to digestion by oral bacteria and thus has little effect on plaque formation and tooth decay [63]. This popular sugar alcohol is also used as an excipient in formulations of various drugs [64] and as a plasticizer [65].

**Scheme 1:**
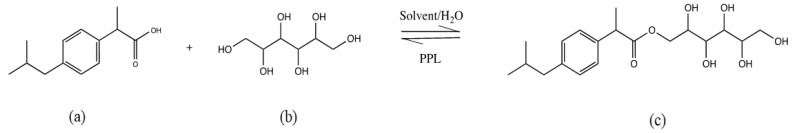
Esterification scheme of (**a**) ibuprofen with (**b**) sorbitol catalyzed by PPL in a biphasic-media environment; PPL: porcine pancreas lipase; (**c**) ibuprofen sorbitol ester.

In the present work, we aimed to modify the chemical structure of ibuprofen by the covalent attack of sorbitol as a hydrophilic compound, exploiting an enzymatic direct esterification (Scheme 1). To this end, free porcine pancreas lipase type II (PPL) was chosen as a biocatalyst as an alternative to the more expensive immobilized enzymes. Sorbitol has previously been used to synthesize a derivative of astaxanthin [66] and one of bixin [67] to improve water solubility. The reference standard was chemically synthesized. Biphasic conditions, such as lipase and compounds concentrations, solvents amount, temperatures, stirring speed, and times are reported here. Finally, the lipase-catalyzed sorbitol ester of ibuprofen (IBU-sorbitol) was chemically characterized by ^1^H, ^13^C-NMR, and MS spectroscopy.

## 2. Results and Discussion

The poor water solubility of ibuprofen characterizes its dissolution-limited oral bioavailability, and this can cause problems in drug tolerability [16].

For the covalent attachment of hydrophilic functional groups to this poorly soluble active ingredient, as a possible strategy for improving its solubility, we focused on lipase-catalyzed esterification of ibuprofen with sorbitol. 

Reactions catalyzed by lipases in non-aqueous media offer new possibilities for the biotechnological production of many useful chemicals using reactions that are not feasible in aqueous media because of kinetic or thermodynamic restrictions [68]. Although immobilized lipases have been shown to display better catalytic activity compared to the non-immobilized lipases in non-aqueous media, we decided to exploit and test the less expensive free form of porcine pancreatic lipase.

To the best of our knowledge, no previous work has proposed enzymatic esterification starting from two solid substrates of such different polarities, nor even the enzymatic synthesis of the prodrug IBU-sorbitol ester. Generally, comparable enzymatic synthesis strategies see the use of one of the two substrates in liquid form, operating both as a reagent and as a solvent, as in the case of liquid alcohol.

Our work has aimed to develop a protocol to obtain effective lipase-catalyzed esterification of ibuprofen with sorbitol using free porcine pancreas lipase (PPL). For this purpose, several factors were considered to establish the operating conditions: the selection of the organic solvent, enzyme concentration, water content, temperature, stirring speed, molar ratio, and reaction time. The conversion yield of the substrate was studied to evaluate the effects of changes in the parameters of the enzymatic reaction.

The reference standard of the sorbitol ester of ibuprofen was chemically synthesized. Subsequently, enzymatic synthesis of IBU-sorbitol ester prodrug was achieved by operating under mild reaction conditions. The purified product was analyzed using NMR and HPTLC-MS.

### 2.1. Chemical Synthesis of the IBU-Sorbitol Ester

Since information on the target compound is not commercially available except for the unknown degree of purity, it was synthesized chemically according to a procedure in the literature [69]. The thusly obtained compound was utilized as a reference compound to allow calibration. The mass spectrometry testifying to the esterification of IBU-sorbitol is reported in the Supplementary Materials (Supplementary Figure S1).

### 2.2. Preliminary Experiment: Selection of a Suitable Organic Solvent

The ability of the free PPL to catalyze the synthesis of IBU-sorbitol ester was investigated, starting with the selection of a suitable organic solvent.

Previously, polyalcohol glycerol was chosen as a hydroxyl group-carrier molecule to increase the polarity of the resulting ester and thus increase its bioavailability [70]. Ibuprofen was esterified with glycerol in a system defined as solventless [47]. In fact, due to its liquid nature, glycerol can act as both a solvent and a reagent. Despite this, the increases in solubility resulting from the covalent attachment of glycerol to poorly soluble water-soluble active ingredients are strongly limited by the only two additional hydroxyls that this polyalcohol can provide following the ester bond. Therefore, to maximize the hydrophilizing effect of adding a polyalcohol to ibuprofen, we decided to choose sorbitol by virtue of its six brought hydroxyls, against the three of glycerol. Although according to this thesis, sorbitol is a better hydrophilic agent than glycerol, its solid nature requires its solubilization in an aqueous medium, so it us able to be exploited solvent-free systems such as those reported for glycerol.

Hence, this lipase-catalyzed esterification needs a biphasic organic solvent/water medium, but not only due to the solid nature of sorbitol. Indeed, although some lipases (such as *Candida antarctica* lipase B) have shown catalytic activity without interfacial activation [71], PPL needs an aqueous–organic solvent interface to catalyze the reverse reaction of synthesis [72,73]. Most lipases, such as PPL, have an α-helical fragment (termed the “lid”) that covers the active site. Conformational change of PPL at the oil–water interface can open the lid, producing lipase activation [74].

In the biphasic system reported in the present work, the organic solvent had a solubilizing role towards the lipophilic compound, ibuprofen, and the water solubilizes the sorbitol polyalcohol. However, finding an adequate organic solvent can be a challenge. Indeed, it is generally reported that solvents with logP < 2 are less suitable for a biocatalytic purpose [75], due to their greatly influencing enzyme activity and substrate specificity [76]. The polarity of organic solvents affects the amount of water in the aqueous layer around the catalyst where the enzymatic reaction occurs, influencing its conversion yield [77]. Thus, based on the logP values of these three solvents, we chose to test hexane, benzene, and toluene as organic solvents. Ibuprofen, due to its strong lipophilicity, proved to be highly soluble in all three organic solvents tested. Furthermore, Ravelo et al. reported solubility tests of ibuprofen in different solvents [78].

LogP is an extensively used parameter to express the polarity of a solvent (or compound) and its possible effect on the enzyme activity in lipase-catalyzed esterification [79]. To determine the effect of the reaction medium on the esterification, IBU-sorbitol ester was synthesized with PPL in these three solvents while we monitored the reactions by TLC and calculated the conversion yields via HPLC (Table 1). 

In a final reactor volume of 15 mL, 20% was water because it is well known that the quantity of water in the reaction medium must be lower than that of the organic solvent to push the lipase towards its synthetic behavior, thereby avoiding the hydrolytic behavior. This amount of water also allowed the complete solubilization of sorbitol.

Interestingly, there appears to be a correlation between the polarity of the organic solvent, its consequent miscibility in water, and the percentual conversion yield. In fact, data of these tests show how the less polar solvent, i.e., hexane, produced a yield of 18%; compare that to the yield of 11% obtained with toluene. Furthermore, with regard to the esterification with benzene medium, this did not give any positive results; in fact, no TLC band attributable to the retention factor associated with IBU-sorbitol ester was found.

This catalytic behavior of the PPL enzyme can be explained by its interfacial activation mechanism. More apolar organic solvents, such as hexane, having limited solubility in water, produce clearer solvent/water interfaces. The better the interface, the better the emulsion produced by agitation, and the greater the space that the lipase has to be able to undergo the phenomenon of interfacial activation and therefore be catalytically active. The dispersion of the organic solvent in water was evaluated. The same organic/water media (solvents only) were analyzed in the same reaction conditions. The presence of organic solvent in water was evaluated after 24 h of controlled agitation and temperature. Spectrophotometric readings allowed us to evaluate how, under these experimental conditions, toluene (260 nm) is 70 times more present than hexane (290 nm) in the aqueous phase separated from the organic one. The greater miscibility of toluene in water than hexane could explain the lower conversion efficiency of lipase in this reaction context, which could suffer the effects of a worse interfacial activation.

Furthermore, the differences in substrate conversion yield may be explained by the capacity of the solvent to stabilize the product IBU-sorbitol. For the reaction to take place effectively, the reaction product needs to be in an organic layer that allows its stability. The nature of the organic solvent strongly influences the enzymatic esterification process, in some cases enough to cancel the ester formation (as it was for benzene). Given that toluene is a more polar solvent than hexane, it would be expected that this can better solubilize the reaction product. However, the data show that the conversion is better, albeit slightly, in hexane. This suggests that the conversion yield is a measure influenced by several factors, which see the concomitant effect on the interface and solubilization of the ester produced. Except for a few literary sources, this ester has not been extensively studied and its distribution mechanisms for organic solvents of different polarity are not thoroughly known. Thus, the purified ester was loaded into biphasic hexane/water and toluene/water media under the same conditions (volumes, temperature, and stirring time) as the enzymatic reaction. The system was stopped, and the partitioning of the ester was evaluated in the organic phase compared to the aqueous one. Only one in ten parts of IBU-sorbitol ester were found in hexane; in contrast, two in ten parts were found in toluene. Thus, due to its higher polarity compared to ibuprofen, IBU-sorbitol ester could split between the organic and aqueous phases in an emulsified biphasic system, while preferentially remaining in the aqueous solvent though. Moreover, we studied the octanol/water partition coefficient (LogP) of the ester of ibuprofen with sorbitol using the shake flask method [80]. IBU-sorbitol ester was found to have a LogP of 1.37 ± 0.09 compared to 3.9 of ibuprofen. This aspect, together with the influence that the solvent has on the interface, may explain the different percentage conversion yields between the solvent hexane and toluene, although slightly. Similar results related to the increment in ester yield in the organic medium were reported in the literature earlier. Been Salah et al. [81] observed that the hydrophobicity of the organic solvent greatly affected and improved the esterification activity. They found higher conversion rates in hexane media. Moreover, Hazarika et al. [82] reported a good correlation between initial esterification rates of PPL and the increase of the hydrophobicity of the solvent.

For this reason, hexane was chosen as the organic medium for further experiments due to higher ester productivity with this solvent. Additionally, hexane has been shown as a suitable organic solvent for the PPL-catalyzed esterification of glycerol and oleic acid [83] and acetylenic acids with n-butanol [84], and for the enzymatic synthesis of ethyl oleate [82].

Furthermore, we evaluated how IBU-sorbitol ester is freely soluble in ethanol and 50% EtOH/water solvent. This could allow evaluating the anti-inflammatory activity of this ibuprofen derivative on cell lines in vitro.

To eliminate the partitioning variable of the product between solvents, these were evaporated before the evaluation of the conversion yield, as reported in the materials and methods section.

### 2.3. Optimization of the Lipase-Catalyzed Synthesis of IBU-Sorbitol

#### 2.3.1. The Effect of Enzyme Concentration

**Figure 1:**
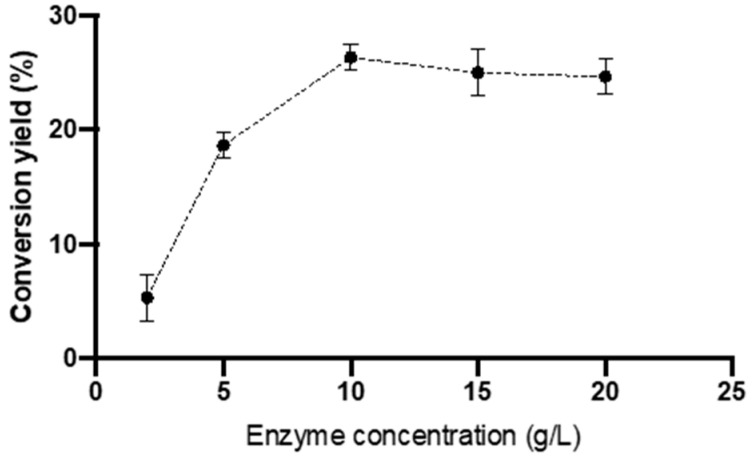
Effect of enzyme concentration on the conversion yield of IBU-sorbitol ester catalyzed by PPL. Reaction time: 15 h (equilibrium condition). All measurements were performed in triplicate.

After the selection of the organic medium, the effect of the concentration of enzyme used was studied and is reported in Figure 1.

To evaluate the effect of enzyme concentration on conversion yield, five concentrations of free PPL enzyme in a range of 2 to 20 g L^−1^ were tested. As shown in Figure 1, the conversion yield increased with the amount of enzyme following a hyperbolic trend typic in the esterification reactions that were lipase-catalyzed. At higher enzyme concentrations, more active sites are present for substrate binding. Hence, the reaction rate increases. However, after 10 g L^−1^, a small decrease in conversion yield is reported. Probably, in a complex system like the biphasic one, since water is present in the system, at high concentrations of enzyme this could hydrolyze the newly formed ester bond.

For this reason, the value of 10 g L^−1^ was selected as the most adequate enzyme concentration for further tests. The enzyme activity of the porcine pancreatic lipase in these conditions has been checked as described in Section 3.3. The results showed the enzyme units were 0.035 ± 0.01 units/mg.

#### 2.3.2. The Effect of Initial Water Content

Handling a biphasic-media esterification can be challenging. Not only is the type of organic solvent chosen decisive, but it is also essential to be able to create a net interface, control the amount of initial water, and be able to determine sufficient agitation for the system to produce an effective emulsion. The volumes of organic solvent and water need to be finely regulated, as do substrate concentrations and the molar ratio.

On this basis, various authors have reported the effect of organic solvents on the performances of lipases. Generally, the hydrophobicity of solvents and the water content are the most important factors influencing the improvement of lipases [85]. Water molecules on the enzyme surface have been described as a molecular lubricant of enzymes [86] and this increases its internal flexibility facilitating the movements necessary for catalysis [87]. Additionally, highly hydrophilic compounds such as sorbitol need a water phase to be solubilized. Indeed, the solubility of sorbitol in organic solvents is too low to reach adequate concentration levels for direct lipase-catalyzed esterification [88]. In contrast, ibuprofen shows higher solubility in non-polar solvents [89]. Despite the organic synthesis with lipases being possible, it must be considered that alcohol and solvents have great effects on the performances of the lipases [90]. 

As for the water, the effect of initial water content on enzymatic activity was examined through the initial addition of an amount of water ranging from 5 to 30 % (*v*/*v*) of the total amount of the reaction mixture (15 mL).

**Figure 2:**
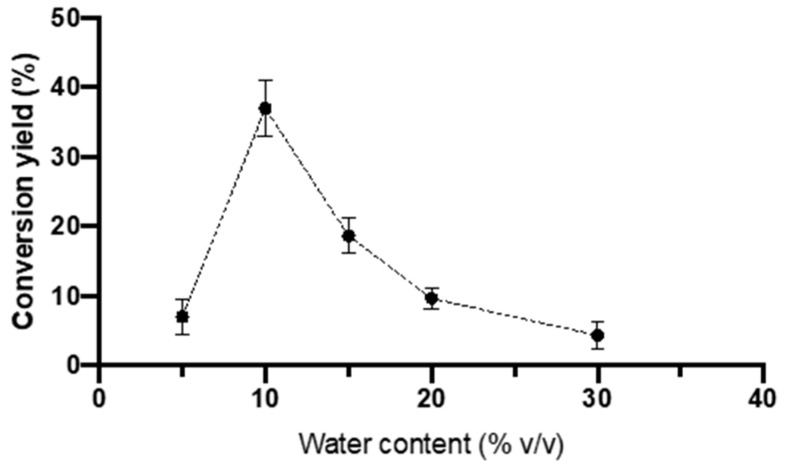
Effect of the amount of water on the ester yield for PPL. Reaction time: 15 h (equilibrium condition). All measurements were performed in triplicate.

The results of Figure 2 show how at low water content (5% *v*/*v*) the activity of pancreatic porcine lipase is strongly limited by the scarcity of the aqueous compartment of the system. Probably, the enzyme is not fully hydrated, resulting in a decreased three-dimensional conformation of the latter. Even more likely, the amount of water corresponding to 5% *v*/*v* is not sufficient to generate and maintain a stable and clear solvent/water interface. This eliminates the interfacial activation effect of the PPL, severely limiting its activity.

At a water content corresponding to 10%, a significant increase in the conversion yield of the substrates was observed. At this amount of water, the lipase is correctly hydrated and manages to be sufficient for the ibuprofen and sorbitol substrates respectively solubilized in hexane and water.

**Figure 3:**
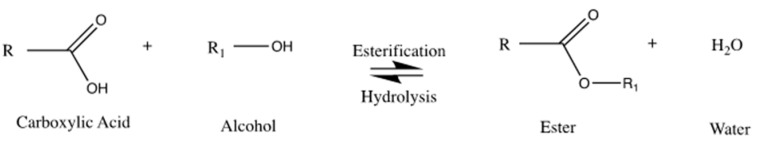
Lipase-catalyzed activity; equilibrium between esterification and hydrolysis.

As the amount of water in the system increases, there is a sharp decline in the conversion yield. This can be explained by the hydrolytic–synthetic balance characteristic of the lipase Figure 3.

Based on this balance, excess water in the system leads the enzyme to produce hydrolysis of the ester bond (as physiologically occurs for the hydrolysis of triglycerides). A similar pattern has been shown in previous studies that report how the excess of the aqueous compartment in the system has destabilizing effects for porcine pancreatic lipase [91].

In a biphasic system such as the one reported in the present work, the reaction medium consists largely of hydrophobic organic solvent, which could determine an accumulation of water near the active site of the lipase—in particular, near-polar amino acid residues. Therefore, the addition of high quantities of water to the system can increase the size of the interfacial area and facilitate the hydrolysis of the ester. Due to this, the production of the ester can be reduced [78,92].

Following this experimental evidence, the value of water content was selected at 10% *v*/*v* for further experiments.

#### 2.3.3. The Effect of Temperature

**Figure 4:**
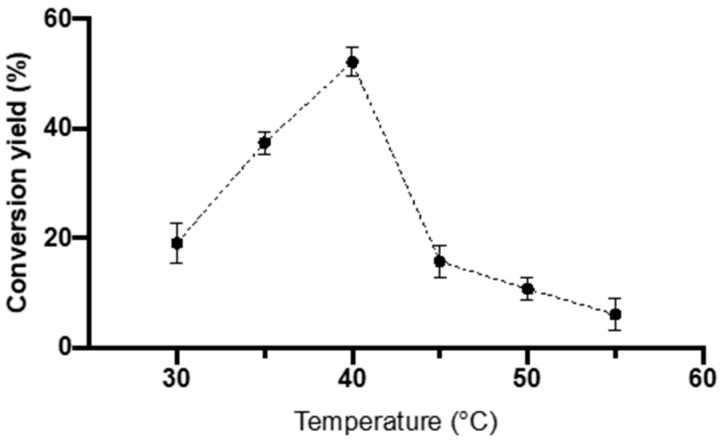
Effect of temperature (°C) on the esterification yield of the IBU-sorbitol ester. Reaction time: 15h (equilibrium condition). All measurements were performed in triplicate.

The effect of temperature on the progress of the PPL-catalyzed esterification reactions was tested at six different temperatures between 30 and 55 °C, and the results are given in Figure 4.

Net changes in the conversion profile of IBU-sorbitol ester resulted from raising the temperature from 30 to 40 °C. The rate of esterification increased enormously with the increase of reaction temperature from 30 to 40 °C, in the absence of catalyst inactivation. However, after 40 °C the ester yield decreased tremendously. Being a biphasic system, the influence of the highly hydrophobic organic solvent is to be considered in the advent of mechanisms related to enzymatic instability. The result shown in Figure 4 supports this thesis. In fact, since PPL is free, or not-immobilized, it is clear that the increase in temperature causes instability of said enzyme, considering the difficult biphasic environment. At higher temperatures, enzymatic instability occurs, which can be observed through visual inspection. The denatured enzyme remains adhered to the walls of the microreactor.

Besides, temperature massively influences the physical characteristics of substrates (solubility, ionization, etc.). The chemical reactivity and the reaction equilibria of the substrate are governed by its thermodynamic properties [93]. However, since this enzyme is in free form, the effect of changes on mass transfer can be considered negligible. Furthermore, when enzymatic esterification involves the use of polyalcohol, the use of low temperatures pushes the enzyme to covalent attack against the primary hydroxyl groups [70]. Other studies reported similar behavior of the porcine pancreatic lipase [94,95].

#### 2.3.4. The Effect of Stirring Speed

Given the highly lipophilic and hydrophilic nature of ibuprofen and sorbitol, respectively, the choice of a biphasic esterification system was obligatory. This is a difficult to manage system with many variables involved. One of the key aspects in managing a medium like the biphasic one is finding the right degree of agitation. In fact, the agitation affects the “interfacial quality” of the interface [96]. Since the reaction medium is a water-in-oil dispersion, the conversion rate would be determined by the interfacial area. The interfacial area of a water-in-oil dispersion determines the conversion rate because it is a function of the speed of agitation and the ratio of the volumes of the aqueous organic phases (in our case the better ratio was 10:1 oil/water). The interfacial area is dependent on the speed of agitation [97]. As this area increases, a greater number of enzyme molecules will become adsorbed onto the interfacial sites. Once all the molecules have occupied sites at the interface, however, any increase in the interfacial area due to an increase in agitation speed will not have an effect.

Conversely, in a system mainly consisting of hydrophobic organic solvent, excessive agitation, added to the administration of heat, can causes enzymatic denaturation [51]. At the same time, if the system is not stirred sufficiently, the emulsion between the organic and aqueous phase is not obtained; this limits the space of the interface where the enzyme can undergo the phenomenon of interfacial activation and thus be catalytically active.

In our case, the choice of the inexpensive PPL enzyme in its free form further complicates matters. In fact, it is reported that the immobilization of enzymes has the effect of increasing their stability against temperature and organic solvents [98,99], an advantage that in this context has disappeared. However, we decided to use a free form of PPL to propose a system that exploits an enzyme without the need for pretreatments, is inexpensive, and is thus highly reproducible. 

**Figure 5:**
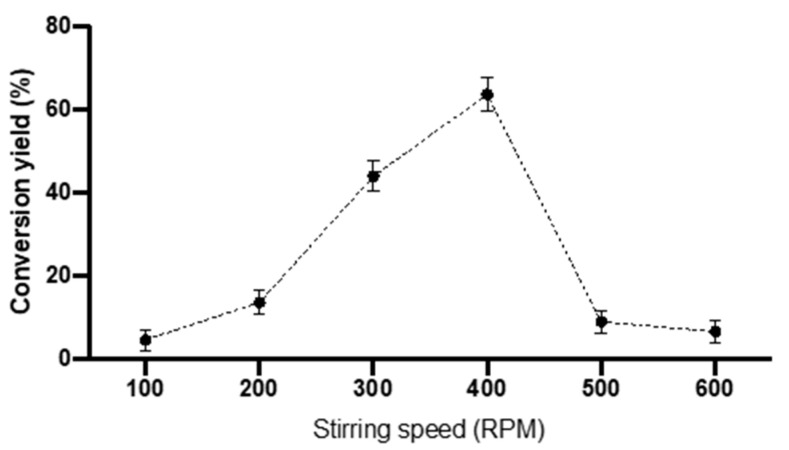
Effect of stirring speed on the enzymatic esterification of ibuprofen with sorbitol. Reaction conditions: PPL = 10 g L^−1^, T= 40 °C, C_IBU_ = 42 g L^−1^, C_Sorbitol_ = 30 g L^−1^, C_w_ = 10% *v*/*v*, volume ratio hexane/water = 10:1. Reaction time: 15h (equilibrium condition). All measurements were performed in triplicate.

By stirring speed [100], we mean the angular speed with which, through the magnetic stirrer, we can produce motion in the system. The effect of the stirring speed in the esterification of ibuprofen with sorbitol in organic/water media was studied by varying the stirring speed between 100 and 600 RPM. These tests allowed us to observe and determine any mass transfer limitation in the biphasic reaction system. The results are shown in Figure 5.

As shown in Figure 5, when enhancing the stirring speed there is an increase in the initial rate of esterification. With hexane being immiscible in the water present in the reaction medium, at low speeds, the contact between the phases is too poor to ensure the meeting of the substrates. The contact between the phases and therefore the meeting between the substrates increases as the agitation increases up to the value of 400 RMP, beyond which, a reasonable decrease in the rate of esterification is observed. The changes in the observation of the esterification rate are attributable, similarly to what has been seen for the increase in temperature, to the phenomena of enzymatic instability. This data are answered by the nature of the free enzyme PPL (in an organic solvent, at 40 degrees), which at an agitation of 500 RPM and up, is very likely to undergo denaturation phenomena. In this system, the optimum stirring speed is reached at which mass transfer limitations and enzyme denaturation can be neglected. Therefore, the value of 400 RMP was chosen for subsequent experimental runs. 

#### 2.3.5. The Effect of Substrate Concentration

Two primary forces determine the acid and alcohol selectivity of lipases: steric hindrance and hydrophobic interactions [101,102]. Testing the effects of acid and alcohol concentrations on the productivity of PPL in catalyzing the esterification reaction of ibuprofen with sorbitol, we first set the alcohol value at 30 g L^−1^ and varied the acid concentrations in a range between 30 and 80 g L^−1^. Secondly, having obtained information on what the best concentration of ibuprofen for enzymatic esterification was, and holding that fixed, we varied the concentrations of polyalcohol sorbitol in a range between 5 and 40 g L^−1^.

**Figure 6:**
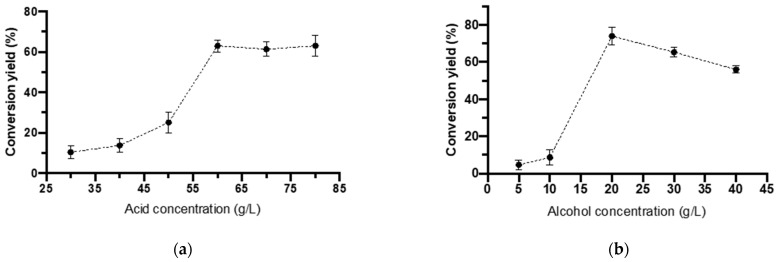
Effects of substrate concentrations on ester yield for PPL; (**a**) 30 g L^−1^ constant sorbitol concentration; (**b**) 60 g L^−1^ constant ibuprofen concentration; reaction conditions: PPL 10 g L^−1^, Cw = 10% *v*/*v*, 40 °C, 400 RPM, 24 h. All measurements were performed in triplicate.

As seen in Figure 6a, when the concentration of ibuprofen increases, there are no inhibitory effects on the enzyme. In any case, given that no further increase in conversion yield is observed beyond the ibuprofen concentration of 60 g L^−1^, this value was taken as a reference for subsequent tests.

After investigating the effects of acid concentration, the alcohol concentration was varied between 5 and 40 g L^−1^. As shown in Figure 6b, the highest yield was obtained at an alcohol concentration equal to 20 g L^−1^.

In this PPL-catalyzed esterification strategy, estimation of how much alcohol can be added to the reaction medium is essential. Indeed, Brockerhoff et al. reported that hydroxyl groups reduce the activity of PPL [73,101]. Moreover, it has been reported that substrates comprising alcoholic functions are a limiting factor in the esterification yield due to the tendency to localize themselves at the interface between the organic solvent and water, stealing space from the enzyme, which therefore cannot undergo interfacial activation and reach the catalytically active form [103]. The literature reports how three main characteristics of the alcohol in question influence the conversion rate of the enzyme: molecule size, hydrophobicity, and solubility. The binding energy, influenced by the size of the molecule carrying the hydroxyl groups, is the force that allows the conversion of the enzyme into its activated form, when the substrate binds to the active site. When the binding energy is low, the lipase is not in its optimal three-dimensional conformation, and the reaction proceeds slowly. Moreover, the more soluble the hydroxyl carrier molecule is in water, the more the enzyme is exposed to alcoholic functions, and the more it undergoes denaturation and therefore inactivation. The great limitations related to the choice of sorbitol as a hydrophilic agent carrying hydroxyl groups have strongly influenced the entirety of the experimental design of the lipase-catalyzed esterification of ibuprofen with sorbitol. First of all, a biphasic system was chosen, wherein there was a good quantity of water capable of solubilizing the sorbitol substrate; secondly, the ratio between acid and alcohol always provided an excess of acid compared to the amount of sorbitol due to its effects on the conversion yield that prevented it from being able to do the opposite.

In our tests, beyond the concentration of 20 g L^−1^, a decrease in the concentration rate of IBU-sorbitol ester was observed. Probably, the high concentrations of polyalcohol sorbitol can be considered as facilitators of protein denaturation by solvating the hydrophobic amino acid residues within the active site and stabilizing the denatured rather than the native conformation [97].

#### 2.3.6. The Effect of Reaction Time

**Figure 7:**
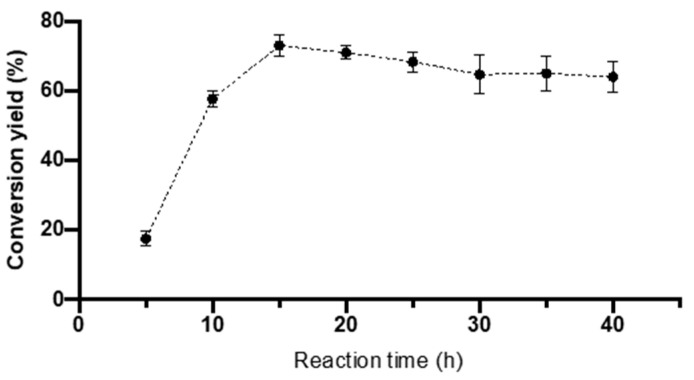
Ester productivity at different reaction times between 5 and 40 h. All measurements were performed in triplicate.

IBU-sorbitol ester synthesis was attained at its optimal conditions at several reaction times between 5 and 40 h, and results are given in Figure 7.

Ester production went up with time for up to 15 h of reaction time and then moderately decreased. The increment of the amount of water produced as a by-product of the catalysis of the ester bond may explain the decrease in conversion yield. Increased water may lead to hydrolysis of the ester previously produced [104]. Furthermore, this decrease may be described also by instability phenomena of PPL in hexane after hours of agitation and heating. Similar results were obtained by Ozyilmaz et al. [105] using PPL for the production of aroma ester.

### 2.4. IBU-Sorbitol Ester: MS Spectroscopy Characterization

Positive electrospray ionization (ESI+) was carried out on the band with an Rf of 0.14 (IBU-sorbitol ester) for the TLC reaction separation of the enzymatic reaction products for mass spectral analysis. The mass spectrometry results confirmed that the esterification reaction took place between the carboxylic acid group of ibuprofen and a hydroxyl group of sorbitol. The esterification product formed had a predicted mass of *m*/*z* 370.

The fragmentation pattern showed in Supplementary Figure S2 (Supplementary Materials) describes the *m*/*z* of the IBU-sorbitol ester as the ionized *m*/*z* 371 [M + H]^+^ adduct (visible both in (a) and (b)) and *m*/*z* 393 [M + Na]+ adduct, confirming the presence of the enzymatically synthesized prodrug. 

To the best of our knowledge, although Douša et al. [69] have chemically produced the ibuprofen ester with sorbitol, this is the first protocol that proposes effective enzymatic esterification of ibuprofen with a highly polar polyalcohol such as sorbitol (with a free enzyme as well). This strategy was demonstrated to be a successful way to produce a prodrug of ibuprofen by direct enzymatic esterification in a biphasic W/O system.

## 3. Materials and Methods

### 3.1. Materials

Free PPL (porcine pancreas lipase, type II; lyophilized (cake); CAS: 9001-62-1, EC: 3.1.1.3) and ibuprofen sodium salt (≥98% pure) were purchased from Sigma-Aldrich (St. Louis, MO, USA). Sorbitol (≥98% pure), N,N′-dicyclohexylcarbodiimide (DCC), 4-dimethylaminopyridine (DMAP), silica gel (60 Å, 70–230 mesh, 63–200 µm), and methanol-d4 degree 99.8% were purchased from Sigma-Aldrich. All other solvents, other than the methanol for HPLC, were of ACS grade. The heated magnetic stirrer was model “AREX Digital PRO,” by Velp Scientifica SRL (Usmate Velate, Italy).

### 3.2. Chemical Synthesis of the IBU-Sorbitol Ester

As reported by Douša et al. [69], for the chemical synthesis of the sorbitol ester of ibuprofen, sorbitol (910 mg; 5 mmol) was dissolved in 5ml of dimethylsulfoxide (DMSO), forming a clear and uncolored solution. Ibuprofen (1.03 g; 5 mmol) and DMAP (0.06 g; 0.5 mmol) were added to this warm solution. Afterward, DCC (1.03g; 5mmol) was added. The mixture was stirred for 20 h and then cooled in liquid nitrogen to solidify it. This solid mixture was placed on lyophilization (Lyovapor L-200; Buchi s.r.l–Italy) to reduce the amount of DMSO. Dichloromethane (15 mL) was added to the mixture, the mixture was cooled down to 5 °C, and the solid was filtered. The filtrate was concentrated in a vacuum at 50 °C to receive 4 g of uncolored oil. 

The result of the reaction was investigated with TLC. The separation was performed with a mobile phase consisting of ethyl acetate/hexane/acetic acid/toluene 60:25:10:5 (*v*/*v*/*v*/*v*). After complete elution, the thin-layer was dried and the separation results were acquired via UV spectroscopy (λ = 254 nm), and after, chemical derivatization with the phosphomolybdic acid solution. For mass spectrometry (MS) and the Rf determination for the IBU-sorbitol ester, the chemically synthesized compounds were analyzed in negative and positive ion mode using an ion trap (Thermo LC-QDuo, Thermo-Finnigan, Waltham, MA, USA). The capillary source voltage was set at 10 V, the source voltage was 4.57 kV, and the capillary temperature was set at 160 °C. Elution of bands was run with the HPTLC-MS Interface (CAMAG) using methanol at a flow rate of 0.1 mL/min.

### 3.3. Enzymatic Synthesis of IBU-Sorbitol Ester

Gardossi et al. [106] described guidelines for reporting biocatalytic reactions. The ester of ibuprofen with sorbitol (IBU-sorbitol ester) was firstly synthesized by testing three different organic solvents to determine which medium was the best for the lipase-catalyzed reaction. The three organic solvents tested for the esterification reaction were: hexane, toluene, and benzene. 

To this end, a ratio of 5:1—solvent/water (water: pH 7; finale volume: 15 mL)—was used to synthesize ester with 5 g L^−1^ of free PPL, along with an acid/alcohol molar ratio of 5:1. Reactions were carried out in screw-capped 20 mL vials immersed in a glycerin bath placed on a magnetic stirrer equipped with a temperature probe capable of self-regulating the temperature of the heating plate to maintain the temperature constant over time (35 °C and 400 rpm). The magnet used as a stirrer for the media in the microreactor was a “double-sided crosshead magnetic stirrer“ of 1.5 cm in diameter.

After 24 h (unless otherwise indicated) of reaction time, the catalyzer was filtered out and the biphasic media was dried with a first rotatory evaporator step (RE-121; Buchi s.r.l–Cornaredo, Italy) and final lyophilization.

For the next investigation steps, the dried substrates were resuspended in methanol. Thin-layer chromatography (TLC Silica gel 60, 5 × 10 cm, Merck, Germany) analysis was used to monitor the reaction progress. The elution system was ethyl acetate/hexane/acetic acid/toluene 60:25:10:5 (*v*/*v*/*v*/*v*). Under these conditions, for the IBU-sorbitol ester, the retention factors (Rf) were 0.14, and the IBU-sorbitol standard was chemically synthesized.

To determine the conversion yield of this preliminary step and all subsequent optimization tests, the conversion yield was calculated by JASCO HPLC modular system equipped with a reverse-phase column (Synergi 4 µm Hydro-RP 80 Å − 250 × 4.6 mm), a refractive index (model RI-4030), and a UV–Vis detector (model UV-4070)—30 °C, mobile phase 73:17 MeOH/H2O (pH 2.2), 0.8 mL/min. The conversion yield was calculated using the following equation:

$$X\;=\;\frac{A_{ester}}{A_{IBU}-A_{ester}}$$

where $A_{ester}$ means area of IBU-sorbitol ester, and $A_{IBU}$ means area of ibuprofen. Negative controls of the reaction were prepared without the use of lipase and all the experiments were conducted in triplicate.

### 3.4. Lipase Activity

PPL activity in hexane was determined by measuring the esterification reaction of ibuprofen by the enzyme using HPLC. PPL (75 mg), ibuprofen (0.20 M), and sorbitol (0.16 M) were equilibrated to a water activity of 1 using atmospheric equilibration over water-saturated potassium sulfate [107]. The mixture was incubated at 37 °C on a shaking water bath (300 rpm). Samples were withdrawn during the reaction for quantification of the decrease of ibuprofen by HPLC (JASCO HPLC with C18 reverse phase Synergi 4 µm Hydro-RP 80 Å − 250 × 4.6 mm equipped with refractive index (model RI-4030) and UV–Vis detector (model UV-4070) [108]. One unit of lipase activity is defined as 1 µmol ibuprofen consumed per min per mg lipase. All measurements were performed at least in triplicate.

### 3.5. Effects of the Esterification Parameters

IBU-sorbitol ester synthesis was carried out with free PPL at various concentrations ranging from 1 to 20 g L^−1^. After determining the best concentration of PPL, the effects of water content in the system were established. In a final fixed volume of 15 mL, the amount of water was tested, ranging from 5 to 30% of water. Subsequently, the effects on the esterification yield of 6 different temperatures were established in a range from 30 to 55 °C. The effect of the stirring speed was evaluated in the range from 100 to 600 RPM, and the effects of acid and polyalcohol concentrations of ester production were examined by two sets of reactions. Primarily, alcohol concentration was kept constant at 30 g L^−1^ and acid concentration was selected between 30 and 80 g L^−1^. The conversion yield was calculated according to the HPLC method described above. Secondly, the same experiment was conducted with different alcohol concentrations between 5 and 40 g L^−1^ optimum acid concentration.

Different effects of reaction time (5–40 h) were studied to find out the best working conditions for IBU-sorbitol ester synthesis.

### 3.6. Purification and Spectroscopic Characterization of IBU-Sorbitol Ester

The reaction products were separated by glass column chromatography. The column was prepared with silica gel using a mixture of ethyl acetate/hexane/acetic acid/toluene 60:25:10:5 (*v*/*v*/*v*/*v*) as elution solvent. IBU-sorbitol ester was isolated. The fractions containing the product were combined, the solvent was evaporated using a rotary evaporator and the residue was analyzed by ^1^H and ^13^C NMR (400- MHz Varian Gemini spectrometer; Varian, Palo Alto, CA, USA). The glass column chromatography allowed us to obtain 1.1 g of ester, with a conversion yield of 67%. The NMR sample was prepared by dissolving it in deuterated methanol (1 mL). ^1^H- and ^13^C-NMR spectra showed that the compound’s structure was the expected one. The characteristic peaks of ibuprofen, attributable to the aromatic ring (zone 7–7.5 ppm) and two methyl peaks (0.85 ppm), were present. Moreover, it can be noted that the signals in the center of the spectrum, in particular, in the 3.5–4.25 ppm zone, are related to the polyol sorbitol.

NMR spectra (provided as Supplementary Materials, Figures S3 and S4) showed the following peaks: ^1^H NMR (400 MHz, CD_3_OD) δ 7.20 (d, J = 8.1 Hz, 2H), 7.08 (d, J = 7.9 Hz, 2H), 4.25 (d, J = 11.4 Hz, 2H), 4.06 (dd, J = 11.4, 6.7 Hz, 1H), 3.95–3.88 (m, 1H), 3.84–3.70 (m, 3H), 3.69–3.53 (m, 2H), 2.46–2.39 (d, J = 7.4 Hz, 2H), 1.83 (m, 1H), 1.45 (d, J = 7.2 Hz, 3H), 0.88 (d, J = 6.6 Hz, 6H); ^13^C NMR (101 MHz, CD_3_OD) δ 175.35 (d, J = 29.8 Hz), 140.29, 138.01, 128.92 (4C), 73.61, 72.08, 71.10, 69.49, 65.58, 63.34, 44.87, 44.62, 30.02, 21.32 (2C), 17.64.

## 4. Conclusions

Ibuprofen is a widely used NSAID limited in bioavailability by its poor water solubility. In this work, we proposed an effective optimized enzymatic process for the production of the enhanced-water soluble IBU-sorbitol prodrug. The direct enzymatic route has been optimized for: biphasic media dynamics, enzyme concentration, water content, temperature, stirring speed, substrates concentrations, and reaction time. ^1^H, ^13^C-NMR, and MS confirmed the PPL-catalyzed esterification of ibuprofen with sorbitol. To the best of our knowledge, this is the first time that enzymatic esterification of IBU-sorbitol ester has been proposed. This process initiates the synthesis with the biocatalytic approach of numerous bioactive molecules with increased bioavailability of pharmaceutical-industrial and agri-food interest.

## Figures and Tables

**Table 1:** Effects of various organic solvents on IBU-sorbitol ester production by PPL.

Solvent	LogP	Solubility in Water	Conversion (%)
Hexane	3.9	9.5 mg mL^−1^	18 ± 1.3
Toluene	2.43	526 mg mL^−1^	11 ± 1.1
Benzene	2.13	1790 mg mL^−1^	-

The reaction conditions were 5 g L^−1^ of free lipase; solvent/water; acid/alcohol ratio 5:1; incubation at 35 °C with stirring at 400 rpm; 24 h.

## Supplementary Materials

The following are available online at https://www.mdpi.com/1422-0067/22/6/3066/s1. Figure S1: HPTLC-mass spectra in negative ionization mode of the chemically synthesized IBU-sorbitol ester (m/z 369). Figure S2: ESI+ HPTLC-MS analysis of lipase-catalyzed esterification of ibuprofen with sorbitol by free PPL in biphasic media. (a) Na+ adduct of IBU-sorbitol ester. (b) H+ adduct of IBU-sorbitol ester. Figure S3: 1H-NMR of IBU-sorbitol. Figure S4: 13C-NMR of IBU-sorbitol ester.
