# Supplementary material for: Biocatalytic Approach for Direct Esterification of Ibuprofen with Sorbitol in Biphasic Media
Source: Int J Mol Sci. 2021 Mar 17;22(6):3066. doi: 10.3390/ijms22063066 (PMC8002397; doi:10.3390/ijms22063066)

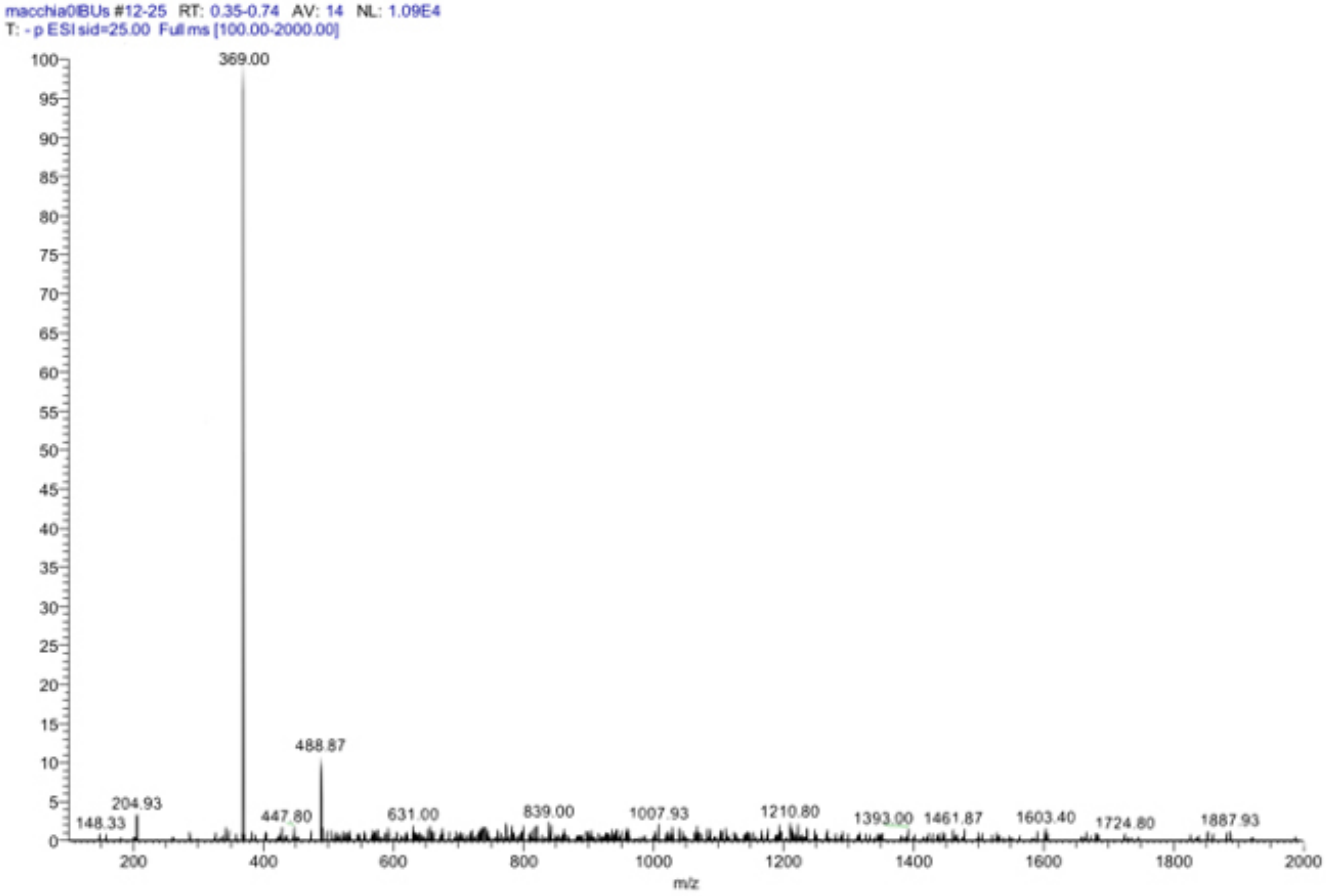

HPTLC-mass spectra in negative ionization mode of the chemically synthesized IBU-sorbitol ester (m/z 369).

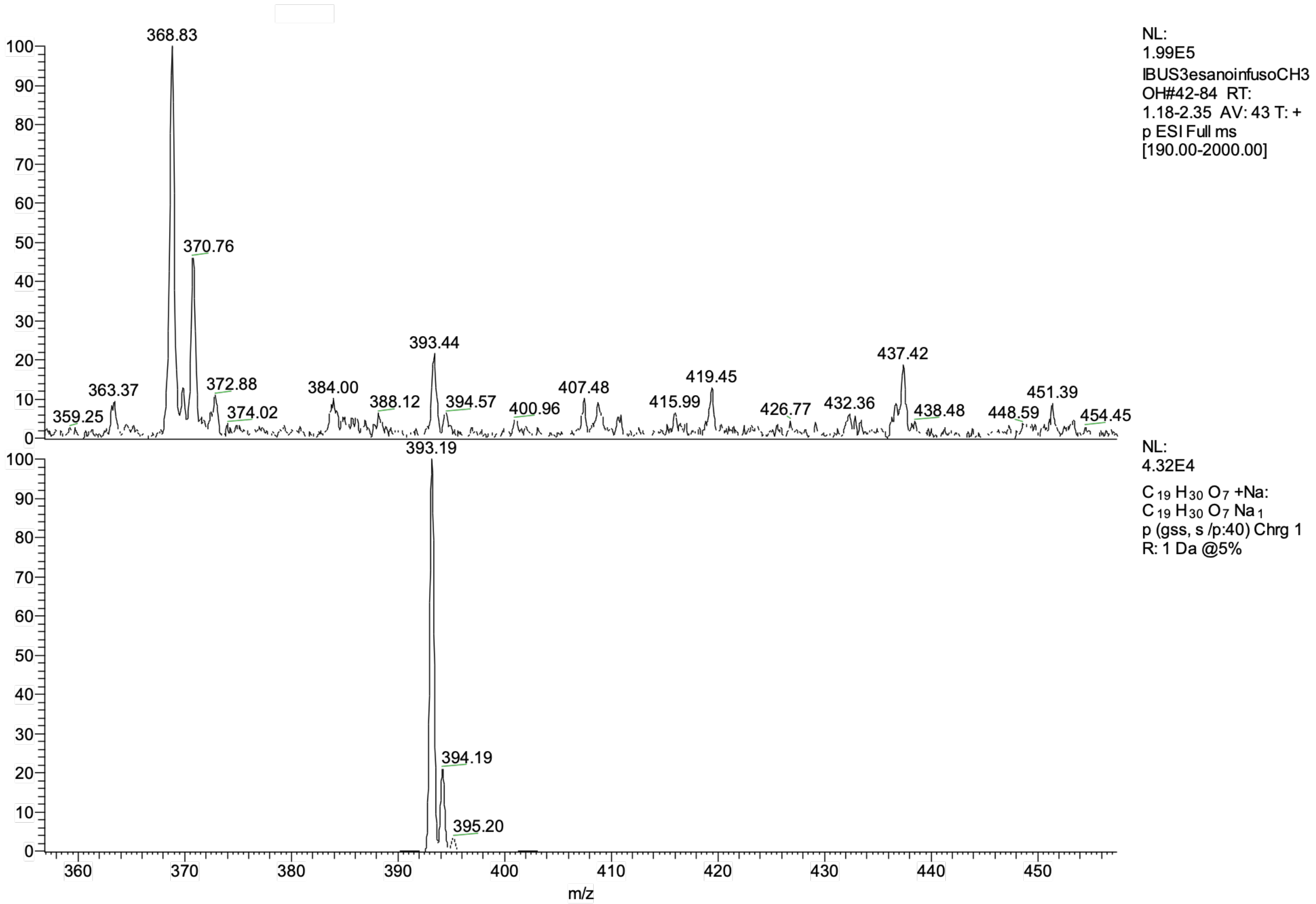

(a)

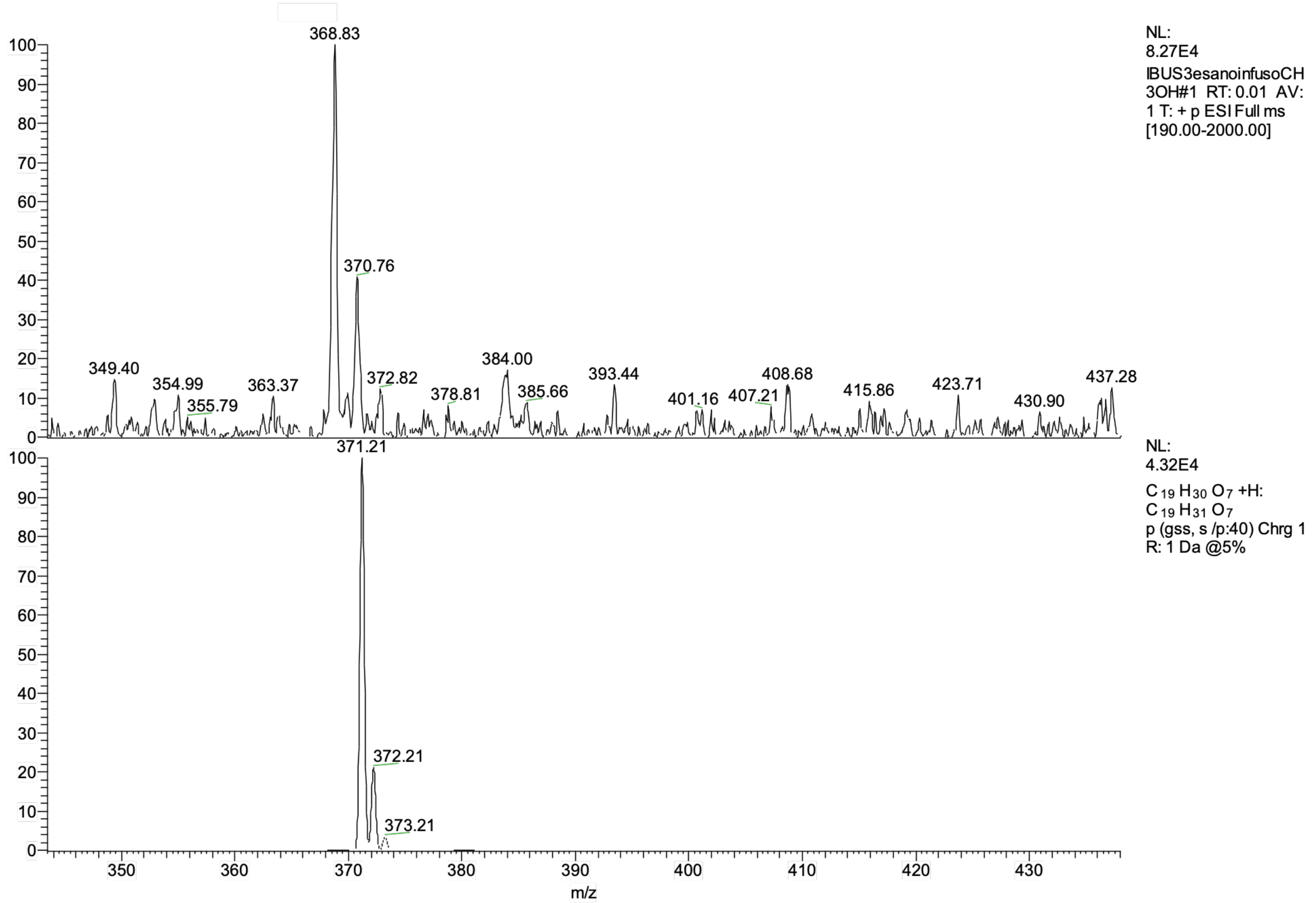

(b)

ESI+, HPTLC-MS analysis of lipase-catalyzed esterification of ibuprofen with sorbitol by free PPL in biphasic media. (a) Na^+ adduct of IBU-sorbitol ester. (b) H^+ adduct of IBU-sorbitol ester.

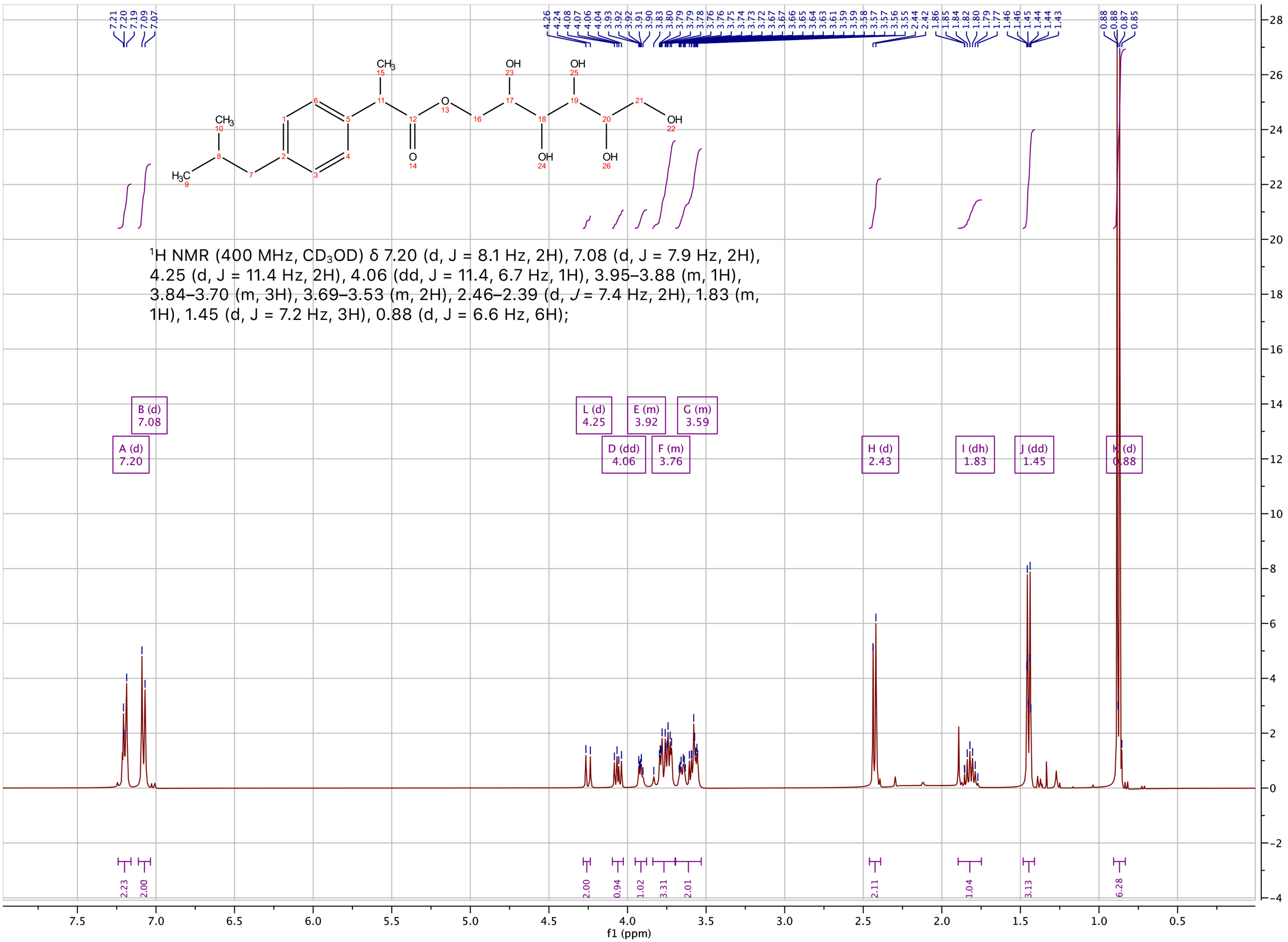

CH3
OH
OH
O
OH
OH
OH
H3C
O
CH3
¹H NMR (400 MHz, CD3OD) δ 7.20 (d, J = 8.1 Hz, 2H), 7.08 (d, J = 7.9 Hz, 2H), 4.25 (d, J = 11.4 Hz, 2H), 4.06 (dd, J = 11.4, 6.7 Hz, 1H), 3.95–3.88 (m, 1H), 3.84–3.70 (m, 3H), 3.69–3.53 (m, 2H), 2.46–2.39 (d, J = 7.4 Hz, 2H), 1.83 (m, 1H), 1.45 (d, J = 7.2 Hz, 3H), 0.88 (d, J = 6.6 Hz, 6H);
A (d) 7.20
B (d) 7.08
L (d) 4.25
D (dd) 4.06
E (m) 3.92
F (m) 3.76
G (m) 3.59
H (d) 2.43
I (dh) 1.83
J (dd) 1.45
K (d) 0.88
2.23
2.00
2.00
0.94
1.02
3.31
2.01
2.11
1.04
3.13
6.28
f1 (ppm)

^{13}C NMR (101 MHz,) δ 175.35, 140.29, 138.01, 128.92, 73.61, 72.08, 71.10, 69.49, 65.58, 63.34, 44.87, 44.62, 30.02, 21.32, 17.64.

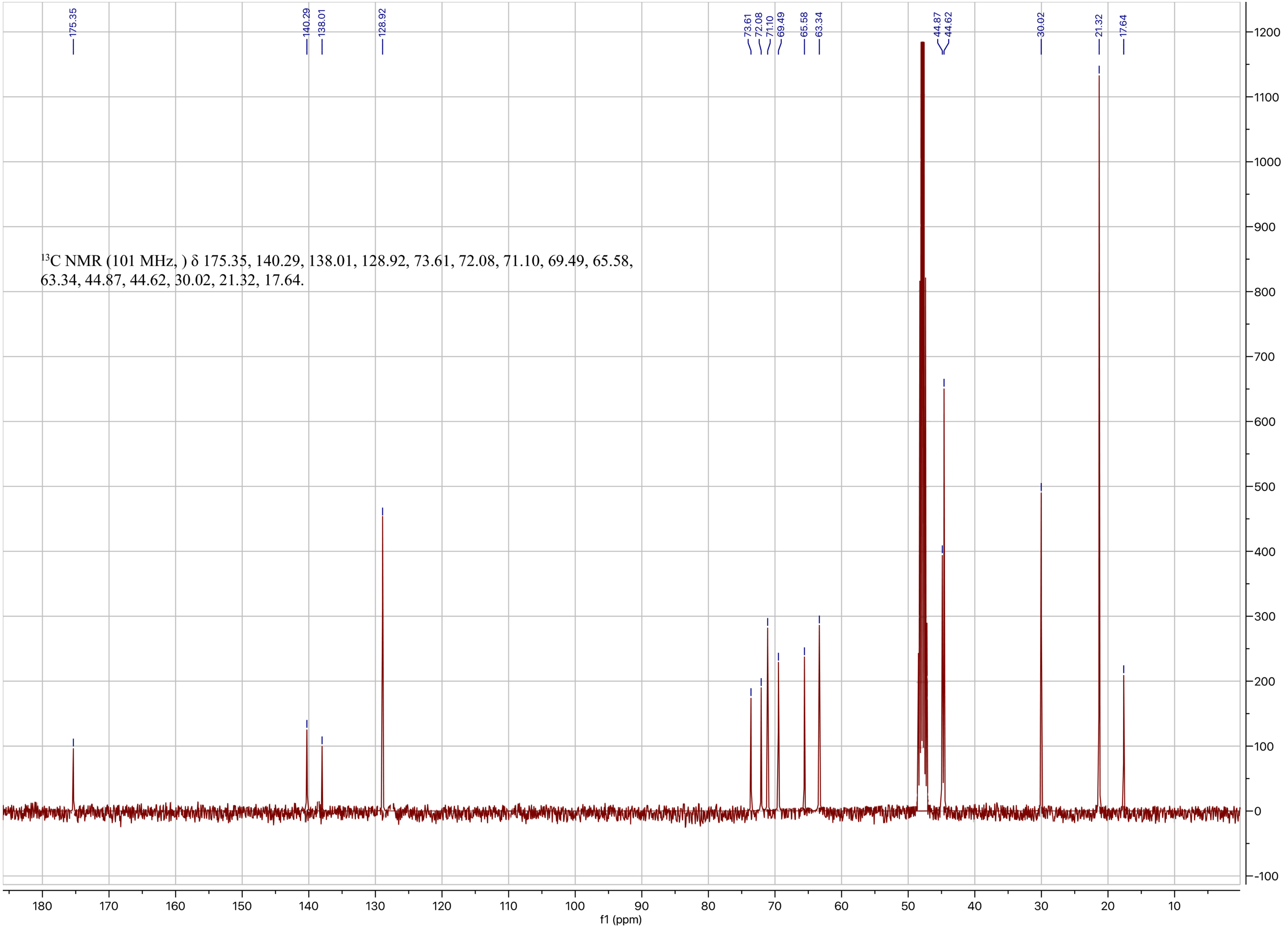

Supplement: Supplementary file 1 [file ijms-22-03066-s001.pdf]
